# Targeting the chemokine receptor CXCR4 for cancer therapies

**DOI:** 10.1186/s40364-025-00778-y

**Published:** 2025-05-01

**Authors:** Ariana Rueda, Naroa Serna, Ramon Mangues, Antonio Villaverde, Ugutz Unzueta

**Affiliations:** 1 Institut de Recerca Sant Pau (IR SANT PAU), Sant Quintí 77 - 79, Barcelona, 08041 Spain; 2 CIBER de Bioingeniería, Biomateriales y Nanomedicina, Instituto de Salud Carlos III, Madrid, 28029 Spain; 3 Josep Carreras Leukaemia Research Institute (IJC Sant Pau), 08041 Barcelona, Spain; 4 Institut de Biotecnologia i de Biomedicina, Universitat Autònoma de Barcelona, 08193 Bellaterra, Spain; 5 Departament de Genètica i de Microbiologia, Universitat Autònoma de Barcelona, Bellaterra, 08193 Spain

**Keywords:** CXCR4, Cancer Stem Cells, Biotechnology, Targeting, Drug delivery, Therapy

## Abstract

The C-X-C chemokine receptor type 4 (CXCR4) has emerged as a key molecular biomarker for cancer therapies due to its critical role in tumor progression and metastases by displaying a stem cells phenotype. Its overexpression has been observed in more than 20 types of cancers, including solid tumors and hematological malignancies, and it is often associated with tumor aggressiveness and poor prognosis. Being initially recognized as a co-receptor involved in HIV infection, numerous CXCR4-targeting ligands and antagonists, including small molecules, peptides and biologics have been identified over the past decades. While only few of them have been used in the context of cancer therapies, recent biotechnological advancements using CXCR4 as a molecular target are showing significant potential to revolutionize future cancer therapies. Therefore, this review highlights the biotechnological innovations developed for cancer therapy and diagnosis by targeting the chemokine receptor CXCR4. It also discusses future perspectives on emerging therapeutic strategies, ranging from the use of small molecule inhibitors that block receptor signaling to cutting-edge nanocarriers designed for the targeted delivery of innovative drugs and proteins into cancer stem cells, aiming at cell-selective precision nanomedicines.

## Introduction

**Fig. 1:**
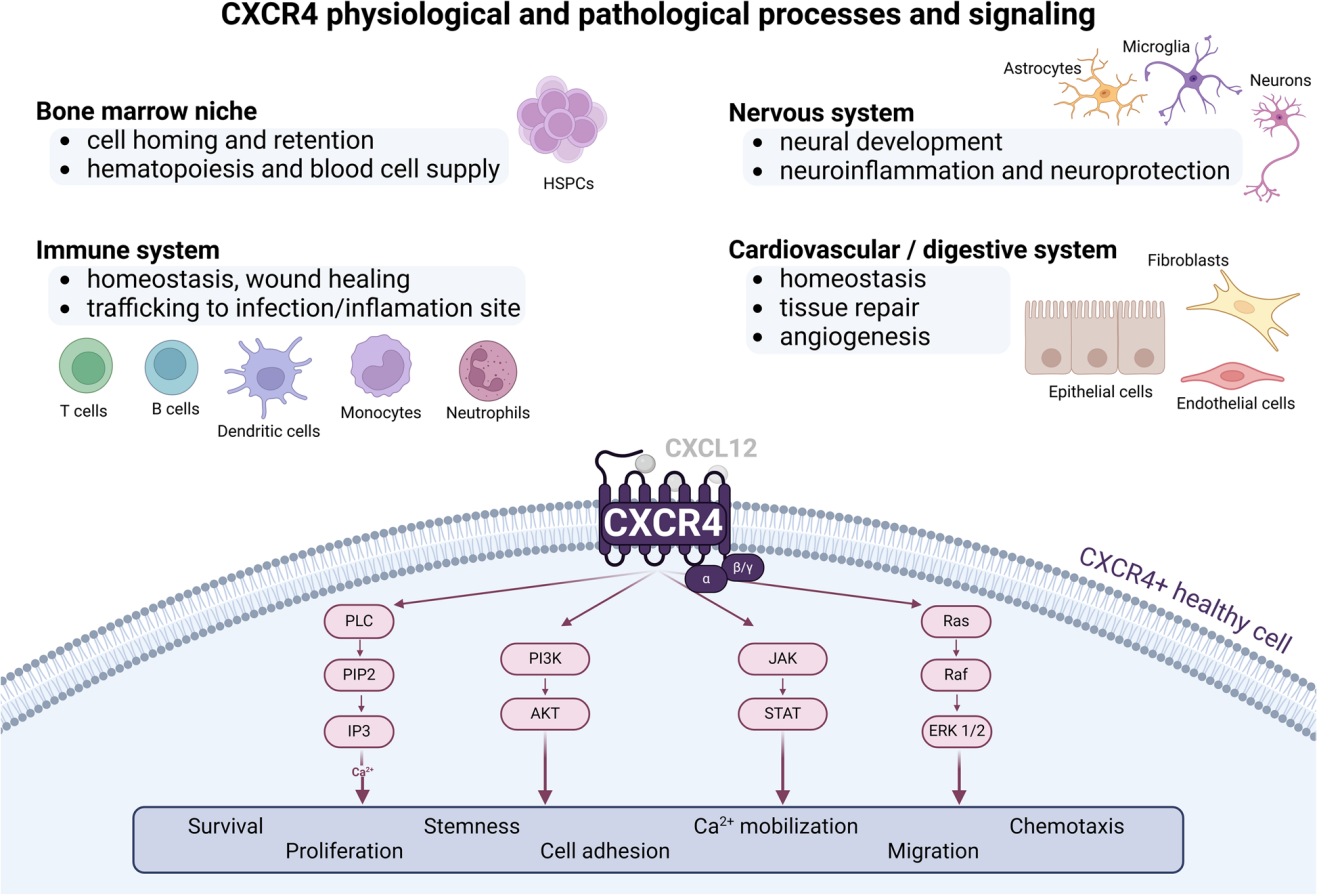
CXCR4 signalling in physiological or pathological processes. The physiological role of CXCR4 is listed for Hematopoietic Stem and Progenitor Cells (HSPCs) in the bone marrow and other cell types on the immune, nervous, cardiovascular and digestive systems. The pathological role of CXCR4 in inflammation, infection and injury repair is also indicated. The CXCR4/CXCL12 interaction and the activated main signalling pathways, namely, PLC, PI3K/Akt, JAK/STAT and MAPK are represented bellow, as well as their downstream cellular responses

The C-X-C chemokine receptor type 4 (CXCR4), also known as CD184, is a membrane protein that contributes to the regulation of cell migration and trafficking of several cell types, particularly involving, among others, immune cells [1–3]. Being widely expressed in diverse tissues and organs, although at different extents, this protein plays crucial roles in numerous physiological functions and several pathological processes. With a typical seven-transmembrane topology, CXCR4 is a G protein-coupled receptor (GPCR) that primarily binds to its natural ligand, the stromal cell-derived factor 1 (SDF-1 or CXCL12) [4, 5]. Such binding triggers structural rearrangements in CXCR4, resulting in its activation [6]. The functional form of CXCR4 then interacts with the Gαi subunit of the heterotrimeric G protein complex, a fact that consequently activates various downstream signaling pathways, including the phosphoinositide 3-kinase (PI3K) pathway, phospholipase C (PLC) pathway, Janus kinase/signal transducers and activators of transcription (JAK/STAT) pathway and the mitogen-activated protein kinase (MAPK) pathway [7, 8]. These pathways collectively regulate processes such as intracellular calcium mobilization, cell adhesion and migration, stem cell homing and immune responses, particularly by stimulating the trafficking of immune cells to sites of infection and inflammation [2, 9–11] (Fig. 1).

In the immune system, CXCR4 is displayed on the surface of T and B cells, dendritic cells, monocytes and neutrophils [12–15]. Interestingly, CXCR4 is a co-receptor used by the human immunodeficiency virus (HIV) during infection of immune cells [16], which has largely contributed to the elucidation of its structure and function. During HIV-linked research, mainly oriented to identify blockers of the viral infection, many CXCR4 agonists and antagonists have been identified [17–20]. Apart from SDF-1 (CXCL12), the CXCR4 natural ligand, the synthetic small molecule AMD3100 (Plerixafor), initially developed for stem cell mobilization [21], has been widely used as antiviral drug [22]. However, many other CXCR4 ligands, including Polyphemusin II and derivatives [23], KRH-1636[24] or FC131 [25]among others [26, 27], have been also identified or developed as antiviral molecules. Despite their medical interest in acquired immunodeficiency syndrome (AIDS)-related pharmacology and other clinical fields, their development for other applications has been limited.

The physiological contribution of CXCR4 has been also recognized in other biological contexts. In the cardiovascular and digestive systems, CXCR4 contributes to homeostasis, tissue repair and angiogenesis [28–31]. In the nervous system however, it is involved in neural development, neuroinflammation and neuroprotection [32–35]. One of the main physiological roles of CXCR4 is its participation in hematopoiesis, assisting the retention of hematopoietic stem cells within the bone marrow microenvironment for the continuous supply of the blood cell catalog [36–39]. Of course, this fact has indirect implications in tissue repair, stress responses, and the prevention of hematological malignancies [40] (Fig. 1).

Apart from its physiological functions, dysregulated CXCR4 signaling is associated with the progression of autoimmune diseases such as rheumatoid arthritis and systemic lupus erythematosus [41–43], as well as in chronic inflammatory conditions like osteoarthritis [44]. Interestingly, CXCR4 is also a relevant molecular marker in cancer, as it is overexpressed in more than 20 human malignancies including the highly prevalent lung, breast, pancreas, ovarian, colorectal, hepatic and prostate cancers, as well as some forms of leukemia and lymphoma [45–48]. CXCR4 overexpression is usually correlated with poor prognosis and often associated with metastasis, that depends on the CXCL12-mediated promotion of cancer stem cells migration and invasion [49]. The very high level of CXCR4 expression in those cancers, compared to healthy tissues, places this receptor as an interesting marker for diagnosis but also, as a potential tool for precise targeting and selective delivery of antitumoral drugs [50–52]. This approach involves displaying specific CXCR4 interactors on drugs or drug carriers, their cooperative CXCR4 cell surface binding, and the consequent endosome-mediated cell internalization [53, 54]. Under the demand of highly selective cytotoxic agents to enhance efficacy and reduce side toxicities in oncological therapies, CXCR4 emerges as a pivotal agent for intervening in several types of malignancies through the potential control of metastatic spread and chemotherapy resistance. This review summarizes different biotechnological approaches and therapeutic strategies under which such objectives has been addressed in the last decades, aiming at precision-oriented drug-based clinical interventions in innovative advanced oncological therapies.

## The chemokine receptor CXCR4 in cancer

The overexpression of the chemokine receptor CXCR4 in cancer cells is primarily regulated by epigenetic mechanisms, including gene hypomethylation in the promoter region and histone acetylation, which is controlled by Histone Acetyltransferases (HATs) and Deacetylases (HDACs). Moreover, non-coding RNAs upregulate CXCR4, as seen with miR-340-5p, which promotes growth and metastasis in colorectal cancer or miR-588, which drives tumor growth in head and neck carcinoma [55]. In addition, the expression of CXCR4 is also increased by the direct effect of transcription factors such as nuclear factor κB (NF-κB), which promotes breast cancer migration and metastasis [56], or the hypoxia-inducible factor HIF-1α, which promotes dissemination in colorectal cancer [57]. Furthermore, chronic hypoxia, resulting from rapid tumor growth and restricted vasculature, stimulates neoangiogenesis through HIF-1α and VEGF, both of which upregulate CXCR4 expression [58].

It is important to note that CXCR4-overexpressing cells (CXCR4^+^) are considered cancer stem cells (CSCs), due to their self-renewal and tumor-initiating capacity in mouse models of different cancer types, properties that differentiated cancer cells (CXCR4^−^) do not display [48, 59, 60]. Moreover, CXCR4^+^ cancer cells show all the characteristics of malignancy, which are high proliferation rate, resistance to conventional therapy, relapse, progression and dissemination from the primary tumor to distant organs.

### CXCR4^+^ cancer stem cells and tumor pathology

CXCR4-overexpressing cells induce tumor proliferation by overactivating the MAPK, PI3K/Akt and PLC signalling pathways. The activation of these pathways also induce drug resistance and relapse by precluding the initiation of cell death after the treatment with conventional chemotherapy or radiotherapy [47, 61]. This effect occurs through the activation of the JAK/STAT pathway, which promotes cell survival by upregulating anti-apoptotic signalling and downregulating the expression of cell death receptors [60]. Additionally, overexpression of immune checkpoints (e.g.CTLA-4, PD-L1, etc.) enables cancer cells to evade the immune system attack [62].

CXCR4^+^ cancer stem cells also show increased tumor invasion and cell migration, via the activation of Rac1 and MMP-2/MMP-14 proteinases and enhanced angiogenesis by triggering cancer cell adhesion to endothelial cells and upregulation of VEGF/VEGFR [63]. This process confers cancer cells the capacity to intravasate, enter the bloodstream, and extravasate, thereby contributing to homing, colonization and metastasis in organs that secrete CXCL12/SDF-1, the ligand of the chemokine receptor CXCR4 [64].

**Fig. 2:**
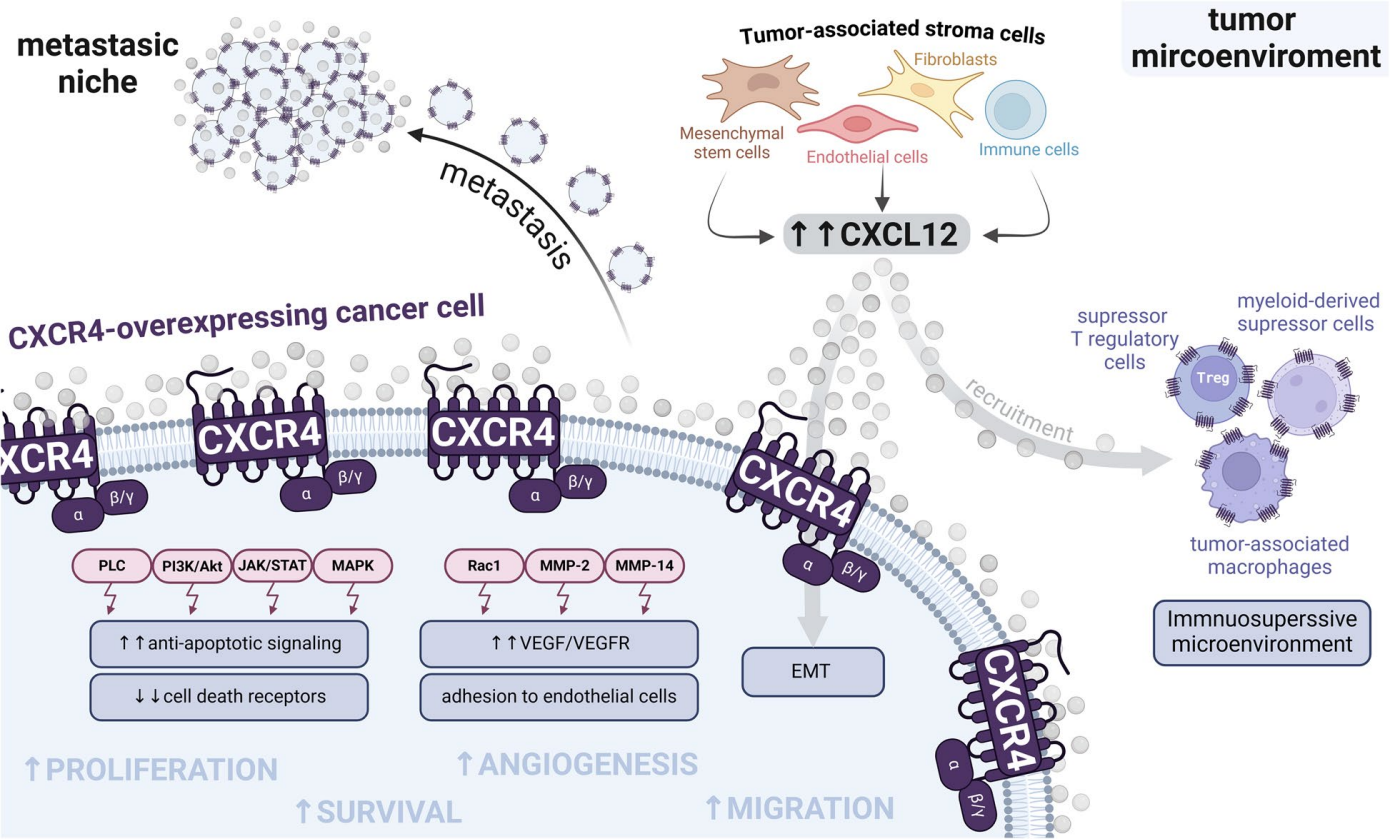
The roles of CXCR4 in cancer. Pathological downstream effects in CXCR4 overexpressing cancer cells due to the aberrant activation of the PLC, PI3K/Akt, JAK/STAT, MAPK, Rac1 and MMP2/14 pathways. At the tumor microenvironment, high levels of CXCL12 secreted by tumor-associated stroma cells contribute to tumor pathology, by activating the CXCR4 signalling in cancer cells, by recruiting CXCR4^+^ immunosuppressive cells, and by inducing epithelial-to-mesenchymal transition (EMT). At metastatic niches, high levels of CXCL12 induce the migration of CXCR4^+^ tumor cells to the metastatic organs

Importantly, the tumor microenvironment (fibroblasts, endothelial cells, immune cells) is also dysregulated by CXCR4^+^ epithelial cancer cells. High levels of CXCL12 secretion by tumor-associated stromal cells is able, in a paracrine way, to enhance cell proliferation, induce epithelial-to-mesenchymal transition (EMT) and increase the migration and invasiveness at the tumor margin [47, 61]. Moreover, CXCL12 secretion promotes tumor infiltration by immunosuppressive regulatory T cells, tumor-associated macrophages, and myeloid-derived suppressor cells, thereby blocking the activation of the immune system against the tumor [64] (Fig. 2).

### CXCR4 overexpression and prognosis

Considering the mechanistic consequences of the features displayed by CXCR4^+^ cancer cells, it is expected that their derived tumors exhibit aggressive behavior. In fact, there is a strong association between CXCR4^+^ tumors and poor prognosis. This is because overexpression of CXCR4, compared to CXCR4^−^ tumors, is linked to poorer outcomes in cancer patients, including an increase in resistance to chemotherapy, higher risk of recurrence, greater metastasis burden, and shorter progression-free and overall survival [45, 60]. Interestingly, among the CXCR4^+^ cancer types there are solid tumors of high prevalence and very poor response to current treatments such as pancreatic cancer, gastric carcinoma and non-small cell lung cancer [47, 60].

## Ligands for CXCR4

In recent decades, a diverse catalog of CXCR4-specific ligands, including both receptor agonists and antagonists, has been developed, each of them offering unique therapeutic potential for cancer treatment and/or targeted drug delivery (Table 1). CXCR4 agonists, such as the natural ligand stromal cell-derived factor-1α (SDF-1α), play essential roles in biological processes like cell migration, tissue repair, and the homing of hematopoietic stem cells. CTCE-0214, a synthetic agonist, is a modified version of the SDF-1α peptide designed to enhance its bioavailability and specificity for CXCR4 activation. This synthetic peptide has been investigated for its potential to promote tissue repair and cell migration more effectively than its natural counterpart [65]. Additionally, ATI-2341, a pepducin, functions as an allosteric agonist of CXCR4, modulating cellular responses by promoting biased signaling between G proteins and β-arrestins, which can lead to distinct therapeutic outcomes [66]. These peptide agonists have been extensively studied in preclinical trials, particularly for their potential in stem cell mobilization and tissue repair. However, their clinical use is less common compared to CXCR4 antagonists due to the complex and often unpredictable effects of CXCR4 activation, which can lead to varied and sometimes adverse responses in different cell types and tissues. Therefore, the main clinical advancements have focused on the development of CXCR4 antagonists, which inhibit CXCR4 activity by blocking its signaling pathways. Given CXCR4’s significant role in cancer progression, these antagonists hold considerable promise as therapeutic agents and targeted drug delivery ligands. They include a range of synthetic and natural peptides, monoclonal antibodies, small molecules, and natural products.

**Table 1:** Ligands for the chemokine receptor CXCR4

Material	Therapeutic agent	Type	Description	Structural features	Main study area	Refence
Small molecule	Plerixafor (AMD3100)	Antagonist	Byciclam derivative	Two cyclohexane rings linked by a phenylene bridge	Cancer therapy (cell mobilization, leukemia, multiple myeloma), pulmonary diseases (silicosis), neuropathic pain, inflammatory bowel disease (colitis), and ophthalmology (retinopathy)	[67, 68]
	Mavorixafor (X4P- 001/AMD070/AMD11070)	Antagonist	Benzimidazol derivative	Benzimidazole and tetrahydroquinoline groups	WHIM syndrome, anti-HIV infection, cancer therapy (leukemia, melanoma, pancreatic cancer)	[69, 70]
	Burixafor (TG-0054/GPC-100)	Antagonist	Synthetic bicyclam	Bicyclic core with modifications to enhance greater affinity for CXCR4	Stem cell mobilization, cancer therapy (leukemia, lymphoma, melanoma), inflammation	[71, 72]
	GMI-1359	Antagonist	Glycomimetic	Glycomimetic designed to inhibit both CXCR4 and E-selectin	Cancer therapy (prostate cancer)	[73]
	USL311	Antagonist	Aromatic heterocycle	Aromatic and heterocyclic ring structure	Cancer therapy (glioblastoma)	[74]
	AMD3465	Antagonist	Synthetic bicyclam, analog of AMD3100	Monomacrocyclic (14-member) ring	Cancer theragnostic	[75]
	IT1t	Antagonist	Isothiourea derivative	Small isothiourea derivative-like molecule	Cancer therapy (breast cancer)	[76]
	MSX-122	Antagonist	Benzimidazole derivative	Heterocyclic core with benzimidazole and pyridine rings	Cancer therapy (breast cancer, gastric carcinoma)	[77]
	KRH-1636	Antagonist	Peptidomimetic	It is a linear molecule bearing an arginine sidechain	Anti-HIV infection	[78]
	KRH-3955	Antagonist	Derived from KRH- 1636	Orally bioavailable compound derived from KRH-1636, with greater anti-HIV-1 activity than AMD3100 and KRH-1636	Anti-HIV infection, cancer therapy	[79]
	BPRCX807	Antagonist	Oxazole derivative	Triazole five-membered ring	Cancer therapy (hepatocellular carcinoma)	[80]
Peptide	Stromal cell-derived factor-1α (SDF-1α/CXCL12)	Agonist	Natural cytokine	Small cytokine that belong to the chemokine family (89 amino acids, 8,5 kDa).It has four conserved cysteine residues that form two disulfide bonds	Stem cell mobilization, chemotaxis, tissue protection, angiogenesis	[4]
	CTCE-0214	Agonist	Synthetic peptide derived from SDF- 1α	Modified SDF-1α peptide for enhanced bioavailability and specificity for CXCR4	Tissue repair, stem cell mobilization	[65]
	ATI-2341	Allosteric agonist	Pepducin	15-mer pepducin	Tissue repair, stem cell mobilization	[66]
	T22	Antagonist	Derived from polyphemusin	18-amino acid peptide with β-sheet structure stabilized by two disulfide bridges	Cancer therapy (colon cancer, leukemia), anti-HIV infection, anti-microbial	[81]
	TW70	Antagonist	Derived from T22	Shortened 14-residue peptide with the C-terminal amide and one disulfide bridge	Anti-HIV infection	[82]
	T134	Antagonist	Derived from T22	TW70 without the C-terminal amide + L-citrulline12	Anti-HIV infection	[82, 83]
	T140	Antagonist	Derived from T134	Substitution of tryptophan with naphthyl-alanine in T134	Anti-HIV infection	[82, 84]
	Motixafortide (TN14003, BTK140, or BL-8040)	Antagonist	Derived from T140	14-amino acid cyclic peptide	Cancer therapy (melanoma, leukemia, carcinoma, lung cancer, prostate cancer), osteoartritis, bronchiolitis	[85–87]
	TZ14004	Antagonist	Derived from T140	T140 with amidated C-terminus	Anti-HIV infection	[82, 88]
	FC131	Antagonist	Derived from T22	Cyclic pentapeptide using the bioactive residues of T140	Anti-HIV infection	[89]
	POL3026 (CVX15)	Antagonist	Polyphemusin II mimetic	β-hairpin protein epitope mimic (PEM)	Anti-HIV infection	[90]
	Balixafortide (POL6326)	Antagonist	CXCR4 domain mimetic	14-amino acid macrocyclic structure	Stem cell mobilization, cancer therapy (prostate cancer, breast cancer)	[91, 92]
	POL5551	Antagonist	CXCR4 domain mimetic	β-hairpin protein epitope mimic (PEM)	Stem cell mobilization, cancer therapy (glioblastoma)	[93, 94]
	CTCE-9908	Antagonist	CXCR4 ligand mimetic	17-amino acid synthetic CXCL12 analog	Cancer therapy (osteosarcoma, prostate cancer, melanoma)	[95]
	Peptide R	Antagonist	CXCR4 ligand mimetic	Cyclic peptide derived from the Ar–Ar-X motif in CXCL12	Cancer therapy	[96]
	LY2510924	Antagonist	Novel synthetic peptide	Small cyclic peptide with non-natural amino acids (propietary sequence)	Stem cell mobilization, cancer therapy (hematological tumors)	[97]
	LY25109249	Antagonist	Novel synthetic peptide	Optimized version of LY2510924, structural modifications (propietary sequence)	Cancer theragnostic	[97]
	LFC131	Antagonist	Novel synthetic peptide	5-amino acid peptide	Cancer therapy	[98]
	E5	Antagonist	Novel synthetic peptide	22-amino acid peptide	Cancer therapy	[99]
Antibody	Ulocuplumab (BMS-936564/ MDX-1338)	Antagonist	Humanized IgG4 monoclonal	Fully humanized IgG4 monoclonal antibody targeting CXCR4	Cancer therapy (hematological and solid tumors)	[100, 101]
	PF-06747143	Antagonist	Humanized IgG1 monoclonal	Humanized IgG1 monoclonal antibody targeting CXCR4	Cancer therapy (hematological tumors)	[101, 102]
	LY2624587	Antagonist	Humanized IgG1 monoclonal	Humanized IgG1 monoclonal antibody targeting CXCR4	Cancer therapy	[103]
	F50067	Antagonist	Humanized IgG1 monoclonal	Humanized IgG1 monoclonal antibody targeting CXCR4	Cancer therapy (hematological tumors)	[104]
	ALX-0651	Antagonist	Single-domain antibody	Biparatopic VHH nanobody	Stem cell mobilization, cancer therapy	[105]
	AD-214	Antagonist	Recombinant Fc-fusion protein	Fc-fusion protein	Fibrotic processes (ILD and CKD)	[106]
Natural Products	Penicillixanthone A	Antagonist	Marine-derived	Flavonoid	Anti-HIV infection	[107]
	Saikosaponin A	Antagonist	Plant-derived, Radix bupleuri	Triterpenoid saponin	Cancer therapy, Inflammation	[108]
Aptamer	NOX-A12 (olaptesed pegol)	Antagonist	Spiegelmer aptame	Single-stranded RNA modified to resist degradation (Spiegelmer)	Cancer therapy (hematological tumors)	[109]
Lipids	BAT1	Antagonist	Cholesterol lipid	A bis(cyclam)-capped cholesterol lipid	Cancer therapy (hematological tumors)	[110]

### Peptides

Peptides derived from a naturally occurring horseshoe crab protein are among the most notable CXCR4 antagonists. One of the key peptides is T22, also known as [Tyr5,12,Lys7]-polyphemusin II, an 18-amino acid peptide derived from the antimicrobial peptide polyphemusin I found in the horseshoe crabs. It adopts an antiparallel β-sheet structure stabilized by two disulfide bridges. This peptide has been extensively studied for its antimetastatic [111], antimicrobial [112], and anti-HIV properties [81]. TW70 is an advanced version of T22 with modifications that enhance its binding affinity and antagonistic activity against CXCR4. It is a 14-residue peptide featuring a C-terminal amide and one disulfide bridge that retains the antiparallel β-sheet structure. It also includes a type II' β-turn with D-Lys8 and Pro9 at the (i + 1) and (i + 2) positions [82]. T134 is a variant of TW70, which incorporates a [L-citrulline12] and lacks the C-terminal amide. This variant shows stronger anti-HIV activity and significantly reduced cytotoxicity compared to TW70 or T22 [82, 83]. Further substituting tryptophan in T134 with naphthyl-alanine results in a peptide with five times higher anti-HIV activity than the original peptide, known as T140 [82, 84]. Interestingly, T140 derived peptides have shown significant promise in cancer therapies. Among them, the 14 amino acids cyclic T140 analogue, Motixafortide (also known as TN14003, BTK140 or BL-8040 from BioLineRx) has been successfully evaluated in preclinical and clinical trials for various CXCR4-overexpressing tumors [85, 86], particularly in combination with checkpoint inhibitors [87]. Additionally, amidation of the T140 C-terminus (TZ14004) has also increased its stability in serum, although this modification also increases cytotoxicity [82]. Finally, structure–activity relationship (SAR) studies on T140 has identified four crucial amino acids (Arg2, Nal3, Tyr5, and Arg14) responsible for its bioactivity. These key residues were used to design novel cyclic pentapeptides, leading to the development of low-molecular-weight CXCR4 antagonists with bioactivities comparable to T140, such as FC131 [89].

Among CXCR4 domain mimetics, POL6326 (known as Balixafortide, developed by Spexis), is an orally bioavailable 14-amino acid macrocyclic peptide that has been investigated for its potential in treating solid tumors such as prostate cancer and HER2-negative metastatic breast cancer, as well as for imaging [91, 92]. POL5551, an analog of POL6326 that differs by only one amino acid, has demonstrated superior efficacy compared to the FDA-approved CXCR4 inhibitor plerixafor. POL5551 has shown promise in preclinical models for targeting glioblastoma stem cells and mobilizing hematopoietic stem and progenitor cells (HSPCs) [93, 94].

Beyond CXCR4 domain mimetics, the β-hairpin mimetic antagonist POL3026 (also known as CVX15), designed from polyphemusin II, has shown beneficial effects as an antiviral therapy in preclinical models [90]. Additionally, the CXCL12 mimetic CTCE- 9908, a cyclic 17-amino acid peptide, has shown promise as a CXCR4 inhibitor [95]. Similarly, Peptide R, a cyclic peptide antagonist derived from the Ar–Ar-X motif in CXCL12, has also demonstrated potential in imaging and drug delivery applications [96].

Finally, among novel CXCR4 antagonist peptides, LY2510924 and its analog LY25109249, both cyclic peptides developed by Eli Lilly, have been investigated for their potential in treating various CXCR4^+^ cancers [97]. Additionally, other novel peptide antagonists widely used in CXCR4 targeted drug delivery include the linear five-amino acid variant of FC131, known as LFC131 [98, 113] and the 22-amino acid peptide E5 [99].

### Small molecules

One of the most widely known CXCR4 antagonists is AMD3100 (also known as Plerixafor), a bicyclam derivative consisting of two cyclam rings linked by a phenylene bridge. Plerixafor has been extensively used for stem cell mobilization and was approved by the FDA in 2008 for autologous transplantation in patients with non-Hodgkin’s lymphoma and multiple myeloma [22, 67, 68]. Another potent analog of AMD3100 is AMD3465, which features a monomacrocyclic structure with a 14-member ring. Due to structural modifications, it exhibits a ten-fold higher affinity for CXCR4 compared to AMD3100 and it has been mainly studied as a theragnostic in preclinical research [75]. Additionally, Burixafor (also known as GPC-100 or TG-0054), is another molecule based on the cyclic bicyclam structure that has shown greater affinity for CXCR4 than AMD3100, with potential in cancer therapy [71, 72].

To address the limitations of macrocyclic structures, several orally bioavailable non-macrocyclic derivatives are being developed. One example is X4P- 001, also known as Mavorixafor or AMD-070, which incorporates benzimidazole and tetrahydroquinoline groups. Mavorixafor has shown clinical promise in treating WHIM syndrome[69], a rare immunodeficiency, and it is also being investigated for cancer therapy [70]. Another promising compound is GMI-1359, developed by Glycomimetics, which inhibit both CXCR4 and E-selectin, showing therapeutic potential through its dual inhibition properties [73].

Additional small molecule inhibitors include USL311, an orally bioavailable compound developed by Proximagen that selectively binds to CXCR4 due to its aromatic and heterocyclic ring structure [74, 114]. Other non-macrocyclic inhibitors such as IT1t (a small isothiourea derivative-like molecule) [76], or heterocyclic ring-containing molecules such as MSX-122 and BPRCX807, are currently in preclinical stages and are being investigated for various therapeutic applications including cancer therapy [77, 80].

Finally, other small molecules in development for CXCR4 antagonism include KRH-1636 [78] and its orally bioavailable derivative KRH-3955, which have shown significantly more potent anti-HIV-1 activity compared to AMD3100, making them promising candidates for anti-CXCR4 therapies [79].

### Monoclonal antibodies

Several monoclonal antibodies (mAbs) targeting CXCR4 have been developed for therapeutic use, particularly in cancer treatment. One example is Ulocuplumab (also known as BMS-936564 or MDX-1338), a fully humanized IgG4 monoclonal antibody developed by Bristol Myers Squibb, which has demonstrated efficacy in preclinical models of both hematologic and solid tumors [100]. Other examples include PF-06747143, a humanized IgG1 anti-CXCR4 antibody from Pfizer [101, 102], LY2624587, a recombinant humanized anti-CXCR4 monoclonal antibody from Eli Lilly [103] and F50067 (Hz515H7), an IgG1 monoclonal antibody developed by Pierre Fabre SA, that have been primarily studied in hematologic malignancies with early-stage clinical trials [104].

In addition to conventional antibodies, advancements in protein engineering have led to the development of novel therapeutic antibody derivatives. For instance, ALX-0651, a biparatopic anti-CXCR4 VHH developed by Ablynx, Inc., has been designed specifically for stem cell mobilization [105]. Another innovative example is AD-214, a recombinant Fc-fusion protein developed by AdAlta. AD-214 consists of two AD-114 i-body molecules that bind to CXCR4 at the front end, fused to an Fc fragment at the tail. This fusion protein has shown safety, tolerability, and favorable pharmacokinetic properties in Phase 1 clinical trials involving patients with interstitial lung disease (ILD) or chronic kidney disease (CKD) and its use is focused on treating fibrotic diseases [106].

### Natural products

Several natural products have been also identified as CXCR4 antagonists. In this regard, Penicillixanthone A (PXA), a marine-derived flavonoid dimer, which acts as a CCR5/CXCR4 dual-coreceptor antagonist with interesting applications in HIV infection [107] and various components from traditional Chinese herbs, such as sakosaponin A (SSA), which have shown potential as CXCR4 inhibitors have been described [108].

### Aptamers and lipids

Emerging approaches targeting CXCR4 include also some aptamers and lipids. A notable example is NOX-A12 (also known as olaptesed pegol), a Spiegelmer aptamer that targets CXCR4 and has been primarily investigated in CXCR4^+^ hematological malignancies [109]. Lastly, a bis(cyclam)-capped cholesterol lipid, known as BAT-1, has been developed for CXCR4-targeted drug delivery systems [110].

## Targeting the chemokine receptor CXCR4 for cancer therapies

### CXCR4 as molecular marker for diagnosis and response monitoring

**Fig. 3:**
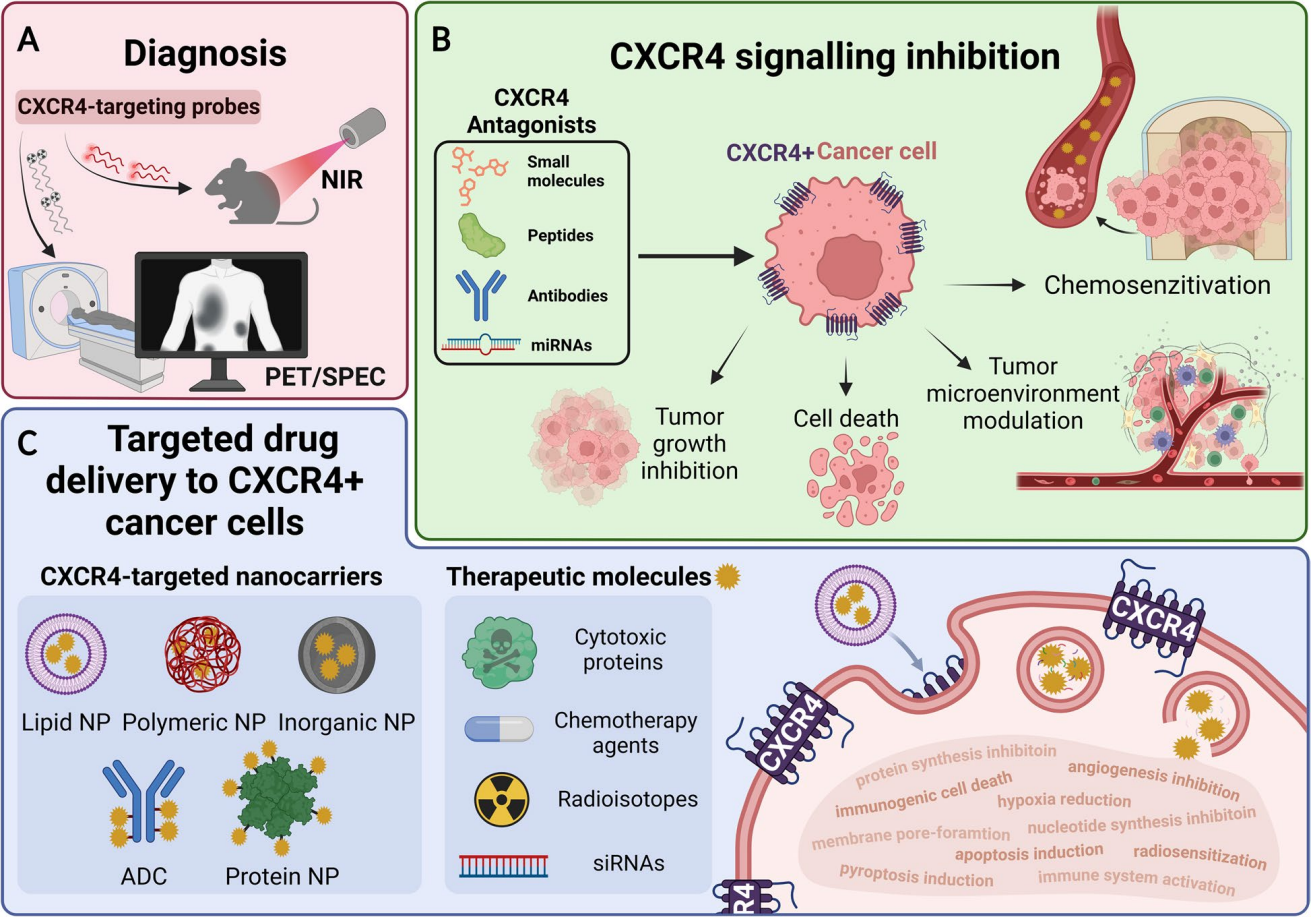
Targeting the chemokine receptor CXCR4 in cancer diagnosis and therapy. **A** CXCR4 as molecular marker for diagnosis and response monitoring using CXCR4-targeted probes in imaging techniques such as positron emission tomography (PET), single proton emission computed tomography (SPECT) or near-infrared (NIR) fluorescence. **B** Inhibitors of CXCR4 for cancer therapy. CXCR4 antagonists, namely small molecules, peptides, antibodies and microRNAs are represented, along with their potential therapeutic effects. **C** Targeted drug delivery to CXCR4^+^ cancer cells. Drug carrier nanoparticles (NP) composed of different materials, loaded with different therapeutic payloads, and their antitumor effect in CXCR4 overexpressing cancer cells. ADC: Antibody Drug Conjugate

The overexpression of CXCR4 in multiple cancer types and its pivotal role in tumour progression via the CXCR4/CXC12 axis makes this chemokine receptor an attractive target for cancer diagnosis, response assessment and patient profiling [114]. Molecular radioimaging offers a non-invasive approach where a targeted radiolabelled tracer is tracked by positron emission tomography (PET) or single-proton emission computed tomography (SPECT) [115, 116] (Fig. 3A).

Pentixafor (CPCR4-2) is a CXCR4-targeting agent that can be labelled with the gallium-68 isotope (^68^Ga-Pentixafor) by the 1,4,7,10-tetraazacyclododecane-1,4,7,10-tetraacetic acid (DOTA) chelator. This radiotracer is considered a breakthrough in PET imaging for haematological cancer diagnostic due to its high selectivity for CXCR4^+^ tumors, minimal hepatic accumulation and high contrast images. It has been successfully evaluated in lymphoproliferative malignancies, offering complementary or superior results to the [18F]-Fluorodeoxyglucose imaging [117]. ^68^Ga-Pentixafor has also been evaluated in chronic lymphocytic leukemia (CLL) [118], non-Hodking lymphoma (NHL) [118, 119], acute myeloid leukemia (AML) [120, 121] and multiple myeloma (MM) [122–126], being the latter the most clinically studied.

Despite its effectiveness in hematologic cancers, Pentixafor has shown more limited use in solid tumors [127–129]. PentixaTec, a Pentixafor derivative tailored for Technetium-99 labelling ([^99^mTc]Tc-PentixaTec) has also exhibited selective uptake in CXCR4^+^ tissue with promising results for SPECT imaging [130]. While other isotopes have also been conjugated to Pentixafor or Pentixafor-based peptides [131], ^68^Ga-Pentixafor remains the most clinically relevant.

In addition to Pentixafor, derivatives of the CXCR4 targeting peptide LY25109249 have also been modified by DOTA conjugation (FRM001) to be labelled with gallium-67/68, Yttrium-90 and Lutetium-177 for radioimaging and potential theragnostic applications. In this sense, ^67/68^Ga-FRM001 showed high tumor uptake in a mouse model but also, notable hepatic accumulation [132]. 1,4,7-triazacyclononane-1,4,7-triacetic acid (NOTA), a more stable chelator than DOTA, was used in another LY25109249 based tracer to be labelled with Copper-64. The resulting ^64^CuNOTA-CP01 exhibited CXCR4 specific tumour targeting for PET imaging, but still considerable liver accumulation [133]. BL01, another LY25109249 derivative, also showed similar results when labelled with gallium-68 and Lutetium-177 [134], but when reengineered to be labelled with fluroidine-18 (18F), using two different strategies (^18^FBL08 and ^18^FBL09), they achieved high-contrast PET imaging in a preclinical model [135].

Beyond the peptide-based tracers, additional small molecules such as a benzenesulfonamide derivative has also been developed as a radioligand for CXCR4-targeted PET imaging. This small molecule, labelled with fluroidine-18, showed effective tumour detection in mouse models [136].

The well-known CXCR4 antagonist AMD3100, has also been radiolabelled with different isotopes such as Copper-64 [137], gallium-67 [138], Zinc-62 [139] and Technetium-99 for PEC/SPECT imaging [140]. Although these tracers showed CXCR4-specific uptake, they also exhibited off-target accumulation in liver, kidneys, and some immune organs in vivo. Then, although gallium-68 labelled AMD3100 tracers were optimized using several linkers and chelators (DOTA or NODA derivative) to improve biodistribution, they still failed to reduce liver and spleen accumulation [141].

AMD3465, an optimized version of AMD3100 [142], exhibited superior CXCR4 specificity as ^64^CU-AMD3465 for PET imaging, in comparison to ^64^Cu-AMD3100, although it still showed considerable uptake in liver and kidneys [143]. However, ^99^mTc-AMD3465 showed lower accumulation in liver for SPECT imaging in a xenograft mouse model [144]. AMD3465 has also been labelled with Cabon-11 as N-[^11^C]Methyl-AMD3465, showing good CXCR4 selectivity in a C6 glioma tumour model. Importantly, the significantly shorter half-life of Carbon-11 (compared to ^64^Cu and ^99^mTc) could also potentially lessen radiation burden in patients [145]. Moreover, AMD3465 has been radiolabelled with fluorine-18, being the most promising candidates ^18^FMCFB (high CXCR4 binding and selectivity) [146], ^18^FRPS-547 and ^18^FRPS-5324, the latter exhibiting superior tumour-to-normal tissue ratios when compared to ^68^Ga-Pentixafor in vivo [147].

Cyclam derivatives of AMD3100 have been also labelled with iodine-131 and bromine-76, with the latter (^76^Br-HZ270) demonstrating high tumour uptake and low background signal for PET imaging in brain cancer, excluding CNS located tumors [148]. Another CXCR4 antagonist, CuCB-Bicyclam, was labelled with copper-64 (^64^Cu-CuCB-bicyclam) and achieved superior affinity and specificity than ^64^Cu-AMD3100 and ^64^Cu-AMD3456 counterparts in vivo [149].

Apart from radioimaging, fluorescent-based techniques are also emerging for CXCR4-targeted diagnostics. For instance, a sulfo-Cy5 labelled Pentixafor tracer (MK007) has allowed CXCR4-targeted fluorescence endoscopy to detect dysplastic lesions in a mouse model of Barrett’s esophagus [150]. In another approach, Balixafortide (POL6326), a CXCR4 peptide antagonist, has also been successfully conjugated with a fluorescent probe to specifically detect breast cancer metastases in sentinel lymph nodes [151].

### CXCR4 as molecular target for cancer therapy

CXCR4 is an attractive target for cancer therapies due to its role in tumour growth, metastasis and shaping the tumour microenvironment [152]. It also facilitates cancer cell homing and invasion of distant organs, which can shelter and promote proliferation of tumour cells in protective niches like the bone marrow [153]. Targeting and blocking CXCR4 disrupts these processes, thereby blocking tumour progression, reducing resistance to chemotherapy, and supporting immunotherapies, making it a valuable focus in the development of anti-cancer strategies [47, 60, 154] (Fig. 3B). Since CXCR4 is overexpressed in more than 20 cancer types, various antagonists, mainly small molecules, peptides and antibodies, have been developed to target this receptor, many of which have reached clinical trials (Table 2). In this section, the most clinically relevant examples will be discussed, along with additional therapeutic strategies aimed at disrupting the CXCR4/CXCL12 axis.

**Table 2:** Therapeutic interventions targeting CXCR4 in cancer (clinical trials)

Material	Therapeutic agent	Combinational treatment	Cancer type	Clinical stage	Enrollment	Status	ID
Small molecule	Plerixafor (AMD3100)	Monotherapy	Brain tumors	Phase 1/2	30	Completed	NCT01977677
		Monotherapy	Pancreatic, ovarian and colorectal	Phase 1	26	Completed	NCT02179970
		Cytarabine/Daunorubicin/G-CSF	Actue myeloid leukeima	Phase 1	36	Completed	NCT00990054
		Bortezomib/Dexamethasone	Multiple myeloma	Phase 1/2	58	Completed	NCT00903968
		Mitoxantrone/Etoposide/Cytarabine	Actue myeloid leukeima	Phase 1/2	52	Completed	NCT00512252
		Rituximab	Chronic lymphcytic leukemia	Phase 1	24	Completed	NCT00694590
		Cemiplimab	Pancreatic cancer	Phase 2	25	Completed	NCT04177810
		Temozolomide	Glioblastoma	Phase 2	21	Active	NCT03746080
		G-CSF	Multiple myeloma	FDA approved	302	Completed	NCT00103662
		G-CSF	Non-Hodgkin lymphoma	FDA approved	61	Completed	NCT01164475
	Mavorixafor (X4P- 001/AMD070)	Ibrutinib	Waldenstrom's macroglobulinemia	Phase 1	16	Completed	NCT04274738
		Nivolumab	Renal cell carcinoma	Phase 1/2	9	Completed	NCT02923531
		Monotherapy/axitinib	Advanced renal cell carcinoma	Phase 1/2	74	Completed	NCT02667886
		Monotherapy/Pembrolizumab	Advanced melanoma	Phase 1	16	Completed	NCT02823405
	Burixafor (TG-0054/GPC-100)	Monotherapy/G-CSF	Multiple myeloma, non-Hodgkin lymphoma or Hodgkin disease	Phase 2	12	Completed	NCT02104427
		Propanol/Propanol + G-CSF	Multiple myeloma	Phase 2	40^a^	Recruting	NCT05561751
	GMI-1359	Monotherapy	Healthy	Phase 1	58	Completed	NCT02931214
		Monotherapy	Breast cancer	Phase 1	4	Terminated (slow enrollment)	NCT04197999
	USL311	Monotherapy	Solid tumors, glioblasoma multiforme	Phase 1/2	26	Terminated (business reasons)	NCT02765165
Peptide	Motixafortide (BL- 8040)	Monotherapy	Healthy	Phase 1	38	Completed	NCT05293171
		G-CSF	Multiple myeloma	Phase 3	180	Active	NCT03246529
		Monotherapy	Healthy	Phase 1	33	Completed	NCT02073019
		Cytarabine	Actue myeloid leukeima	Phase 2	42	Completed	NCT01838395
		Pembrolizumab/liposomal irinotecan	Pancreatic cancer	Phase 2	80	Completed	NCT02826486
		G-CSF	Multiple myeloma	Phase 3	180	Active	NCT03246529
		Pembrolizumab	Pancreatic cancer	Phase 2	18	Completed	NCT02907099
		Nelarabine	T-Acute lymphoblastic leukemia/​ lymphoblastic lymphoma	Phae 2	12	Termianted (low accrual)	NCT02763384
		Monotherapy	Advanced hematological malignancies	Phase 2	50	Completed	NCT02639559
		Cemiplimab + Gemcitabine + Nab-paclitaxel	Pancreatic adenocarcinoma	Phase 2	10^a^	Recruting	NCT04543071
	LY2510924	Idarubicin + Cytarabine	Acute myeloid leukemia	Phase 1	36	Completed	NCT02652871
		Carboplatin + Etoposide	Extensive-stage small cell lung carcinoma	Phase 2	90	Completed	NCT01439568
		Durvalumab	Advanced refractory solid tumors	Phase 1	9	Terminated after phase 1a was completed	NCT02737072
		Sunitinib	Metastatic renal cell carcinoma	Phase 2	110	Terminated (insufficient efficacy)	NCT01391130
	Balixafortide (POL6326)	Monotherapy	Advanced hematological malignancies	Phase 1/2	38	Completed	NCT01413568
		Monotherapy	Multiple myeloma	Phase 2	21	Completed	NCT01105403
		Monotherapy	Healthy	Phase 1	27	Completed	NCT01841476
		Eribulin	Metastatic breast cancer	Phase 1	54	Completed	NCT01837095
		Eribulin	HER2-negative metastatic breast cancer	Phase 3	432	Terminated (failure to meet the primary endpoint)	NCT03786094
	PTX- 9908 (CTCE- 9908)	Monotherapy	Hepatocellular carcinoma	Phase 1/2	50^a^	Recruting	NCT03812874
Antibody	Ulocuplumab (BMS- 936564)	Monotherapy/Lenalidomide + ​Dexamethasone/Bortezomib/​ Dexamethasone	Multiple myeloma	Phase 1	46	Completed	NCT01359657
		Monotherapy	Acute myelogenous leukemia and B-cell cancers	Phase 1	96	Completed	NCT01120457
		Cytarabine	Acute myeloid leukemia	Phase 1/2	70	Terminated (slow accrual, change in business objectives)	NCT02305563
		Nivolumab	Pancreatic and small cell lung cancer	Phase 1/2	61	Terminated (lack of efficacy)	NCT02472977
		Ibrutinib	Waldenstrom's macroglobulinemia	Phase 1	13	Terminated (sponsor decision)	NCT03225716
	LY2624587	Monotherapy	Advanced cancer	Phase 1	56	Completed	NCT01139788
	PF- 06747143	Monotherapy/Cytarabine + Daunorubicin + Azacitidine + Decitabine	Acute myeloid leukemia	Phase 1	8	Terminated (sponsor prioritization)	NCT02954653
	F50067 (Hz515H7)	Monotherapy/Lenalidomide + Dexamethasone	Multiple myeloma	Phase 1	14	Terminated (toxicity)	[104]
Nanobody	ALX- 0651	Monotherapy	Healthy	Phase 1	52	Terminated (POP established with completed SAD)	NCT01374503

*G-CSF* Granulocyte colony-stimulating factor, *POP* Proof of principal, *SAD* Single Ascending Dose

^a^Estimated

#### Small molecules

One of the most extensively studied small molecule antagonist of CXCR4 is AMD3100, also known as Plerixafor (name Mozobil). It was approved by the FDA in 2008 as a treatment to mobilize HSPCs to the peripheral blood in patients with difficulties for stem cell collection. In this sense, AMD3100 is administered in combination with the granulocyte-colony-stimulating factor (G-CSF) to enhance HSPCs mobilization for autologous transplantation in patients with lymphoma or multiple myeloma [155, 156] and children with lymphoma or solid tumors [157, 158].

Plerixafor is also a chemosensitizer, as its role in stem cell mobilization allows increased exposure of cancer cells to chemotherapy in haematological malignancies such as acute myeloid leukemia (AML) [159, 160] chronic lymphocytic leukemia (CLL) [161], multiple myeloma (MM) [162, 163] and myelodysplastic syndrome (MDS) [164]. It is also being explored in solid tumors, often in combination with immune checkpoint inhibitors like the anti-PD1 Cemiplimab, which has reached phase 2 clinical trials in patients with metastatic pancreatic cancer (NCT04177810). Additionally, Plerixafor has the ability to cross the BBB, and has demonstrated safety in high-grade glioma patients when combined with anti-VEGF therapy [165]. It is also currently being tested with standard temozolomide chemo-radiotherapy in glioblastoma (GBM) patients in a Phase 2 clinical trial (NCT03746080). Overall, plerixafor is the most studied CXCR4 antagonist, with more than 150 registered clinical trials, with its main therapeutic focus in haematological cancers.

Mavorixafor (X4P-001, AMD070) is another orally bioavailable CXCR4 antagonist that enhances CD8^+^ cell infiltration and decreases immunosuppressive cells in the tumor microenvironment. It has been tested in combination with immune checkpoint inhibitors (ICI), such as nivolumab, in patients with advanced renal cell carcinoma (RCC) [70] and axitinib [166], showing manageable safety profiles and potential antitumor activity. Therefore, this could become a triple combination treatment in the future. Mavorixafor has also proved to be clinically safe in combination with Ibrutinib (kinase inhibitor) in patients with Waldenström’s macroglobulinemia [167] and pembrolizumab (anti PD-1) in melanoma [168]. Recently, Mavorixafor has been approved by the FDA for the treatment of WHIM syndrome under the brand name Xalremdi [169].

Burixafor (GPC-100, TG-0054) is another bioavailable CXCR4 antagonist that mobilizes hematopoietic stem cells (HSCs) both in mice and in healthy patients [71]. In a Phase 2 pilot study, Burixafor combined with G-CSF successfully mobilized HSCs into the peripherial blood in patients with non-Hodking lymphoma (NHL), Hodgkin lymphoma (HL) and MM [170]. A novel combinational with beta-adrenergic blocker propranolol and G-CSF showed superior HSCs mobilization compared to standard perixafor plus G-CSF treatment [171]. This triple combination treatment is under study in an ongoing Phase 2 clinical trial in MM patients (NCT05561751).

GMI-1359, developed by GlycoMimetics, is a dual inhibitor of CXCR4 and E-selectin that promotes leukemic cells mobilization to peripheral blood in AML and extended survival in PDX models [19], as well as in preclinical models of MM [172]. In addition, GM1-1359 exhibited anti-metastatic activity in bone and sensitized cancer cells to docetaxel more effectively than single treatment with the CXCR4 antagonist CTCE-9980 [73]. Importantly, GMI-1359 is safe to use in humans (NCT02931214) and has demonstrated on target effect in patients with HR^+^ metastatic breast cancer (NCT04197999).

Finally, USL311, developed by Proximagen, started a phase 1/2 clinical trial alone and in combination with lomustine in patients with advanced solid tumors and recurrent GBM, but was terminated due to business strategy (not safety concerns) (NCT02765165).

#### Peptides

Peptides represent another class of promising CXCR4 antagonist in cancer therapy. Among them, Motixafortide (BL-8040) is one of the most clinically advanced products, representing a potent long lasting CXCR4 antagonist that induces CD34^+^ cells mobilization as a monotherapy [173]. It has received FDA approval under the brand name of Aphexda to be used in combination with G-CSF for HSPCs mobilization and subsequent autologous transplantation in MM patients. This product demonstrated good safety profile in the GENESIS phase 3 clinical trial [174]. Beyond stem cell mobilization, Motixafortide is being explored in combination therapies with cytarabine for relapsed/refractory AML [175] and with nelarabine for relapsed/refractory T-cell acute lymphoblastic leukemia and lymphoblastic lymphoma [176]. Moreover, in combination with both, the PD-1 inhibitor pembrolizumab and chemotherapy, Motixafortide is being evaluated as a second-line treatment in metastatic pancreatic ductal adenocarcinoma (PDAC) [87, 177]. A similar phase 2 study is also currently ongoing to evaluate its effectiveness in combination with the PD-1 inhibitor cemiplimab and chemotherapy, in PDAC patients (NCT04543071). Finally, Motixafortide is being tested as a stem cell mobilizer for gene therapy in sickle cell diseases when combined with Natalizumab [178].

Another peptide antagonist, LY2510924 (Eli Lilly), has entered multiple clinical trials for patients with solid tumors. This peptide initially demonstrated safe CD34^+^ mobilization in a phase 1 dose-escalation study [179] but failed to show significant efficacy improvement in combination with chemotherapy (carboplatin/etoposide) in small cell lung cancer [180] or sunitinib in metastatic renal cell carcinoma [181]. A more recent phase 1 study confirmed its safety in combination with Durvalumab for advanced refractory solid tumors [182], although no further studies have been done. In haematological cancers, preclinical studies have shown that LY2510924 has superior antileukemia activity than AMD3100 in AML as monotherapy and synergistic antitumoral effect in combination with chemotherapy, by efficiently mobilizing leukemia cells, inducing myeloid differentiation and blocking pro-survival signals [183]. Finally, LY2510924 was also safely combined with Idarubicin/Cytarabine for relapsed/refractory AML patients [184].

Balixafortide (POL6326), developed by Spexis, is a peptide antagonist that shows good safety profile when combined with eribulin in HER2-negative metastatic breast cancer [92]. However, this combination did not enhance the performance of eribulin [185]. On the other hand, Balixafortide is able to induce HSPC mobilization as monotherapy in both healthy individuals [186, 187] and patients with haematological diseases (NCT01413568) [188]. Moreover, Balixafortide enhanced the antitumor activity of docetaxel in a murine model of prostate cancer bone metastases [189].

Finally, CTCE-9908 is a CXCL12 analog that strongly blocks the CXCR4/CXCL12 axis, leading to reduced tumor growth and metastases in various preclinical models including osteosarcoma and melanoma [190], esophageal cancer [95], breast [191, 192] and prostate cancer [193, 194]. This peptide, renamed as PTX-9908 by TCM biotech, has advanced in human studies to treat liver cancer. In this sense, a phase 1/2 clinical trial is currently evaluating PTX-9908 after transcatheter arterial chemoembolization (TRACE) to prevent tumor recurrence in patients with non-resectable hepatocellular carcinoma (NCT03812874). Moreover, TCM Biotech is also investigating its use in combination with immune checkpoint inhibitors.

#### Antibodies

Monoclonal antibodies (mAbs) targeting CXCR4 have shown promising results in both solid tumors and hematological malignancies by blocking the CXCR4/CXCL12 axis and inducing cancer apoptosis.

Ulocuplumab (BMS-936564), developed by Birstol Myers Squibb, is a fully human IgG4 monoclonal anti-CXCR4 antibody. Preclinical studies showed antitumor effect in several hematological cancers (AML, NHL and MM) [195] by apoptosis activation via a caspase-independent mechanism that involves the production of reactive oxygen species (ROS) [100]. This antibody exhibits high response rates when combined with lenalidomide and bortezomib in relapsed/refractory MM patients, including those who were heavily pretreated [196]. Additionally, Ulocuplumab has proved to be safe in combination with ibrutinib in patients with mutated Waldernstörm macroglobulinemia [197]. However, a phase 1/2 trial in combination with cytarabine for AML patients was terminated due to slow accrual and change of business objectives (NCT02305563). Similarly, another trial in patients with pancreatic and small cell lung cancer was also terminated because of a lack of efficacy (NCT02472977).

LY2624587 is another fully humanized monoclonal antibody developed by Eli Lilly that promotes apoptosis in preclinical models of hematologic malignancies [103]. This antibody downregulates CXCR4 from the cell surface through receptor internalization although it exhibits weak HSCs mobilization in both animal models and in human studies; especially when compared to its peptide counterpart LY2510924 [103, 198].

PF-06747143, a humanized IgG1 CXCR4 antibody from Pfizer, exhibits strong antitumor activity in preclinical models of NHL, AML and MM [20, 101]. Moreover, PF-06747143 demonstrated strong synergy when combined with bortezomib in a MM and with daunorubicin and cytarabine in chemotherapy-resistant AML PDX models [101]. Unlike other mAbs such as Ulocuplumab, this antibody shows a dual effector mechanism, including antibody-dependent cellular cytotoxicity (ADCC) and complement-dependent cytotoxicity (CDC), in addition to mobilizing malignant haematological cells. PF-06747143 was evaluated in a phase 1 trial with AML patients as monotherapy or in combination with chemotherapy (NCT02954653) but the study was terminated due to strategic business priorities.

Another IgG1 anti-CXCR4 antibody, F50067 (Hz515H7), also reached phase 1 clinical trials based on its preclinical antitumor activity in AML and MM. Similar to PF-06747143, F50067 performs its antitumor effect via ADCC and CDC in addition to hematological cell mobilization[199]. Unfortunately, the clinical studies in relapsed/refractory MM patients receiving F50067 as monotherapy or in combination with lenalidomide and dexamethasone were terminated due to hematological toxicities such as thrombocytopenia [104].

In addition to traditional antibodies, a biparatopic antibody like ALX-0651, considered to be a nanobody, was also explored. Despite its preclinical potential, ALX-0651 did not improve the standard of care treatment in clinical trials (NCT01374503).

#### Oligonucleotide based agents

Multiple miRNAs that regulate CXCR4 expression have been identified to show potential for cancer therapies [200]. Some of them include miR-146, which has shown to supress cell proliferation in colorectal cancer cells (CRC) by downregulating CXCR4 [201]. Additionally, miR-193-5p acts as a tumour suppressor in CRC [202] and enhances the inhibition of CXCR4 when combined with 5-fluorouracil (5-FU) and oxaliplatin, reducing chemoresistance [203]. In breast cancer stem cells, overexpression of miR-139 has shown to diminish stem cell homing and invasion by reducing CXCR4 levels [204]. Similarly, the overexpression of miR-613 supresses the growth and pulmonary metastasis of osteosarcoma by targeting CXCR4 [205]. These facts suggest that miRNAs that regulate CXCR4 could be used to inhibit metastasis and tumor progression in multiple cancers. However, although many tumor suppressor or oncogenic miRNAs targeting CXCR4 have been identified [200], their clinical applications have been hampered mainly due to oligonucleotide degradation in the bloodstream and off-target toxicities [206]. These challenges also apply to other oligonucleotide-based therapeutics such as interference RNAs (RNAi) or small interfering RNAs (siRNAs). Therefore, current efforts are focused on developing suitable nanocarriers or other advanced delivery systems to enhance their stability and target cells specificity [207].

### Targeted drug delivery to CXCR4^+ ^cells

Cancer therapy increasingly focuses on developing innovative treatments that selectively target tumor cells, aiming at minimizing the severe side effects associated with the off-target drug accumulation observed in conventional chemotherapies. In this context, innovative nanomedical tools targeting the chemokine receptor CXCR4, show great promise in selectively eliminating receptor-overexpressing cancer cells, thereby limiting disease progression and tumor dissemination while minimizing side toxicities. Although there are currently no FDA-approved CXCR4-targeted drug delivery systems for cancer, significant efforts are being made in the scientific community to develop such systems due to their huge potential to revolutionize advanced cancer therapies.

With the recent advancements in nanobiotechnology, several non-viral carriers have been developed over the past decades using lipid, polymeric, inorganic, metal, or protein-based materials, aiming at selectively delivering different types of attached or encapsulated drugs into CXCR4^+^ cells (Fig. 3C). The catalogue of delivered molecules include a huge variety of cytotoxic, anti-mitotic and anti-angiogenic drugs as well as small interfering nucleic acids, photothermal molecules or even radioactive isotopes for radiotherapy. Moreover, many of these approaches also report a dual effect, in which the specific blockade of CXCR4/CXCL12 signalling synergizes with the action of the selectively delivered therapeutic molecule (Table 3). Given the promising results observed in preclinical studies, the most successful approaches are expected to move into early-stage clinical trials in the near future.

**Table 3:** CXCR4-targeted drug delivery systems

Material	Nanocarrier	targeting ligand	Therapeutic molecule	Targeted Therapy	Tumor model
Lipid Nanoparticles	Liposomes	SDF1α	Yessotoxin	Chemotherapy	Prostate cancer, breast cancer
		Cyclic peptide R	Doxorubicin	Chemotherapy	Melanoma
		LFC131 peptide	Sorafenib, perfluorohexane, PLX3397	Angiogenesis inhibition, hypoxia reduction, immune activation	Hepatocarcinoma
		AMD3100	VEGF siRNA	Angiogenesis inhibition, CXCR4 blockade	Hepatocarcinoma
		AMD3100	IR780	Photothermal therapy, CXCR4 blockade	Breast cancer
		BAT1	Doxorubicin	Chemotherapy, CXCR4 blockade	Lymphocytic leukemia
	Lipid nanobubble	AMD070	Paclitaxel	Chemotherapy, ultrasound imaging	Breast cancer, cervical cancer
	Membrane derived hybrid nanovesicle	SDF1 analog	Doxorubicin, manganese	Chemoterapy, immunotherapy	Colon cancer, breast cancer
Polymer nanoparticles	PLGA nanoparticles	LFC131 peptide	Doxorubicin	Chemotherapy	Breast cancer, lung cancer
		LFC131 peptide	Epirubicin	Chemotherapy	Hepatocarcinoma
		LFC131 peptide	Sorafenib, metapristone	Angiogenesis inhibition, CXCR4 downregulation, CXCR4 blockade	Hepatocarcinoma
		CTCE peptide	Sorafenib, AZD6244	Angiogenesis inhibition, MEK inhibition	Hepatocarcinoma
		CTCE peptide	p53 mRNA	p53 upregulation, immunotherapy	Hepatocarcinoma
		AMD3100	Sorafenib	Angiogenesis inhibition, CXCR4 blockade	Hepatocarcinoma
		AMD3100	GFP siRNA	GFP knockdown	Breast cancer
	co-polymer micelles	E5 peptide	Doxorubicin	Chemotherapy, CXCR4 blockade	Breast cancer
		E5 peptide	Doxorubicin	Chemotherapy, CXCR4 blockade	AML
	Redox responsive micelles	T22 peptide	Venetoclax, sorafenib	BCL-2 inhibition, FLT3 inhibition	AML
	PAMAM dendrimer	LFC131 peptide	Doxorubicin	Chemotherapy, CXCR4 blockade	Breast cancer
	Chitosan nanoparticle	LFC131 peptide	Doxorubicin	Chemotherapy	Lung cancer
	Dextrin nanogels	AMD3100	Doxorubicin	Chemotherapy, CXCR4 blockade	Breast cancer
	Polycation nanoparticles	AMD3100	RUNX1 siRNA	RUNX1 knockdown, CXCR4 blockade	AML
		AMD3100	miR-200c	EMT blockade, CXCR4 blockade	Cholangiocarcinoma
	Small molecule	Pentixather	Lutetium-177, Yttrium-90	Endoradiotherapy	Multiple myeloma
	Dendrimer	CXCR4L peptide	Lutetium-177, C19	Endoradiotherapy, chemotherapy	Pancreatic cancer
Inorganic nanoparticles	Mesoporous silica nanoparticle	T22 peptide analogue	Doxorubicin	Chemotherapy	B-cell NHL
	Prussian blue nanoparticle	E5 peptide	Daunorubicin	Chemotherapy, CXCR4 blockade	AML
Antibodies	Full Antibody	Anti-CXCR4 IgG	Auristatin	Chemoterapy	Osteosarcoma
		Anti-CXCR4 mAb	Auristatin	Chemoterapy	Lung cancer
		Anti-CXCR4 mAb	Gold nanoparticle	Radiosensitization	Breast cancer
		Anti-CXCR4 mAb	Iron nanoparticles	Magnetic hyperthermia	T-cell leukemia
		Anti-CXCR4 mAb	QD/doxorubicin	Chemotherapy	Multiple myeloma
	ScFv	anti-CXCR4 ScFv	miR-127	CXCR4 blockade, immunotherapy	Breast cancer
Protein nanoparticles	Multivalent nanoconjugates	T22 peptide	5-FdU	Chemotherapy	CRC
		T22 peptide	5-AraC	Chemotherapy	AML
		T22 peptide	Auristatin	Chemotherapy	AML, DLBCL
	Multivalent nanoproteins	T22 peptide	BAK, BAX, PUMA	Pro-apoptotic	CRC
		T22 peptide	Mellitin, gomesin, CLIP71	Pore-formation	Cervix cancer
		T22 peptide	PE24, DITOX	Prot. synthesis inhib	CRC, DLBCL, AML, HNSCC, endometrial cancer, melanoma
		T22 peptide	Ricin	Prot. synthesis inhib	AML
		T22 peptide	GSDMD, MLKL	Pro-pyroptotic, immunotherapy	CRC
	Peptide polyplex	SDF1 derived peptide	VEGFA siRNA	Angiogenesis inhibition	Glioblastoma
	BSA nanocarrier	AMD3100	Paclitaxel	Chemotherapy CXCR4 blockade	Ovarian cancer

*CXCR4L peptide* Cyclo-D-Tyr-D-[NMe]Orn(DOTA-HYNIC)-Arg-Nal-Gly, *MMD hybrid nanovesicle* Macrophage Membrane derived hybrid nanovesicle, *QD* Quantum dot, *BSA* Bovine Serum Albumin, *AML* Acute myeloid leukemia, *CRC* Colorectal cancer, *DLBCL* Diffuse large B-cell lymphoma, *M. Myeloma* Multiple myeloma, *HNSCC* Head and neck squamous cell carcinoma, *NHL* Non-Hodgkin lymphoma, *PE24* De-immunized catalytic domain of *Pseudomonas aeruginosa* exotoxin A, *DITOX* Translocation and catalytic domains of the diphtheria toxin from *Corynebacterium diphtheria, GSDMD* Gasdermin D, *MLKL* Mixed Lineage Kinase Domain-Like protein

#### CXCR4-targeted lipid nanoparticles

Several liposomes have been surface-decorated with CXCR4-specific peptide agonists and antagonists to selectively deliver encapsulated antitumoral molecules to CXCR4^+^ cancer cells. In one approach, pH-sensitive nanoliposomes were covalently decorated with the SDF1α protein, the natural ligand of CXCR4, to encapsulate and target the marine-derived drug Yessotoxin into CXCR4^+^ prostate and adenocarcinoma cells [208]. Similarly, doxorubicin encapsulating liposomes, decorated with the CXCR4 antagonist cyclic peptide R, significantly reduced the effective doxorubicin dose and lung metastases in a mouse model of melanoma [209].

Expanding on this approach, another innovative formulation encapsulated a combination of three drugs, including the angiogenesis inhibitor sorafenib, the hypoxia reducing perfluorohexane and the immune-activating PLX3397 in a single CXCR4-targeted liposome functionalized with the LFC131 peptide. This strategy successfully overcame sorafenib resistance through the synergistic action of the three molecules in CXCR4^+^ patient-derived hepatocarcinoma xenograft models [210].

The well-known CXCR4 antagonist AMD3100 (Plerixafor) has been also extensively used for the surface modification of CXCR4-targeted liposomes. One study employed AMD3100-decorated liposomes to deliver VEGF siRNAs, achieving effective antiangiogenic therapy in hepatocellular carcinoma models [211]. In another approach, the hydrophobic molecule IR780, which has photothermal properties, was encapsulated in AMD3100-decorated liposomes. This system achieved significant anti-tumor and anti-metastatic effects in breast cancer mouse models upon near-infrared laser irradiation [212]. In both cases, AMD3100 served a dual purpose, facilitating intracellular delivery of the therapeutic molecules while simultaneously blocking CXCR4 signalling to enhance the therapeutic effect.

Furthermore, the CXCR4 bis(cyclam) ligand BAT1 has been used for the targeted delivery of doxorubicin to CXCR4^+^ chronic lymphocytic leukemia model, exhibiting a similar dual action mechanism [213]. In a theragnostic approach, lipid nanobubbles with ultrasound contrast properties were conjugated with the CXCR4-antagonist AMD070 and the chemotherapeutic drug paclitaxel. These nanobubbles provided dual functionality for ultrasound-guided tumor imaging and targeted drug delivery [214].

Finally, in an innovative technique, CXCR4-targeted hybrid nanovesicles were developed by combining M1 macrophages-membrane-derived vesicles with liposomes conjugated with an SDF-1 analogue. Generated hybrid nanovesicles were then loaded with manganese and doxorubicin and demonstrated dual functionality for targeted drug delivery and M0-to-M1 macrophage reprogramming. This innovative approach showed potent tumor-suppressing activity in mouse models of colon and breast cancer [215].

#### CXCR4-targeted polymer nanoparticles

Different types of polymeric materials have been used to develop CXCR4-targeted nanoparticles, with poly (lactic-co-glycolic acid) (PLGA)-derived carriers being among the most extensively explored. One approach employed the CXCR4-antagonist LFC131 pentapeptide to decorate the surface of doxorubicin-encapsulating PLGA nanoparticles, achieving targeted drug delivery to breast and lung cancer cells [98, 216]. This peptide was also used for the targeted delivery of epirubicin into hepatocellular xenografts models using similar PLGA-TPGS nanoparticles [217].

Advancing on this approach, a strategy involving the use of LFC131-decorated PEG-PLGA nanoparticles was developed for the co-delivery of the antiangiogenic agent sorafenib and metapristone. This combination resulted in a synergistic effect in hepatocarcinoma xenograft model, as the CXCR4 downregulation induced by metapristone, along with CXCR4 blockage from LFC131, counteracted the upregulation and activation of CXCR4 observed after prolonged sorafenib administration [218]. A similar approach used CTCE peptide decorated PEG-PLGA nanoparticles for the co-delivery of sorafenib and the MEK inhibitor AZD6244, aiming to prevent sorafenib resistance in hepatocellular carcinoma animal models [219, 220]. These nanoparticles were later adapted to deliver p53 mRNA to induce p53 gene expression in hepatocellular carcinoma cells and reduce immunosuppression [221]. Additionally, CXCR4 antagonist AMD3100 has been also incorporated into PLGA nanoparticles for targeted delivery of sorafenib [222] or siRNA molecules [223] into cancer cells.

Other polymeric materials have been also explored. For example*,* the CXCR4-antagonist peptide E5 has been used to target PEG-PE copolymer nanoparticles [99, 224] or DSPE-mPEG2000 micelles [225] to CXCR4^+^cancer cells. These systems achieved significant prolongation of survival in mouse models of breast cancer and AML by combining the blockade of CXCR4 signalling with the targeted delivery of encapsulated doxorubicin. More recently, redox-responsive, disulfide crosslinked polymeric micelles, decorated with the CXCR4-selective peptide T22, were designed for the targeted co-delivery of the BCL-2 inhibitor venetoclax and FLT3 inhibitor sorafenib. This dual inhibitor strategy demonstrated strong synergy, significantly prolonging survival in a mouse model of FLT3-ITD AML [226].

LFC131 peptide has been also used to decorate PAMAM dendrimers [227] or chitosan nanoparticles [113] for the targeted delivery of encapsulated doxorubicin or docetaxel into CXCR4^+^ cancer cells. Additionally, AMD3100 was incorporated into dextrin nanogel nanoparticles for the selective delivery of doxorubicin, enhancing antimetastatic effect in an orthotopic breast cancer model [228]. Interestingly, fully AMD3100-derived polycationic materials have been recently developed that, when coupled with antitumoral siRNA [229] or microRNA molecules [230], simultaneously inhibit CXCR4 and deliver the therapeutic RNAs, offering cooperative dual-action therapies.

Finally, in an innovative approach, the CXCR4-specific PET imaging agent ^68^Ga-Pentixafor has been modified to incorporate different α- and β-emitters, including lutetium-177 (^177^Lu-Pentixather) and yttrium-90 (^90^Y-Pentixather), for the first CXCR4-targeted endoradiotherapy. Combined with conventional chemotherapy and autologous stem cell transplantation, this therapy achieved remarkable effects in patients with advanced multiple myeloma [231]. More recently, a dendrimer nano-radio-vehicle, containing lutetium-177 and encapsulated KRAS membrane association blocking molecule C19, was developed for a dual-targeted radio and chemotherapy in CXCR4^+^ pancreatic cancer cells [232].

#### CXCR4-targeted inorganic nanoparticles

The use of inorganic materials for CXCR4-targeting remains a relatively underexplored approach. Among the few reported strategies, one example involves the development of doxorubicin-loaded mesoporous silica nanoparticles (MSM) capped with a CXCR4-specific T22 analogue peptide. These nanoparticles were designed to selectively internalize and release the cargo drug into CXCR4^+^ B-cell NHL showing effective drug delivery and tumor targeting [233]. More recently, a dual targeting system was developed by combining the CXCR4-especific E5 peptide with hyaluronic acid, to create daunorubicin-loaded Prussian blue nanoparticles. This system, that targets both CXCR4 and CD44 receptors, achieved significant inhibition of leukemia blast proliferation and metastatic dissemination in an AML xenograft model [234].

#### CXCR4-targeted antibody drug conjugates

CXCR4-specific antibodies and their derivatives have not only been used for receptor blockade therapies, but also for targeted delivery of antitumoral molecules in form of antibody–drug conjugates (ADCs). Related to that, one of the first CXCR4-targeted ADC was developed by conjugating a CXCR4-specific IgG to the microtubule inhibitor Auristatin though oxime ligation. This ADC selectively eliminated CXCR4-overexpressing metastatic cells both in vitro and in vivo, achieving full inhibition of tumor growth in a lung-seeding cancer model, while causing a modest effect on non-target CXCR4^+^ hematopoietic cells at the bone marrow [235].

Further optimization of Auristatin-conjugated anti-CXCR4 ADCs focused on lowering its affinity to preserve HSPCs while maintaining tumor-selective cytotoxicity. This approach successfully eliminated CXCR4^+^ cancer cells in solid tumor xenograft models, inducing potent antineoplastic effect while minimizing leucocytosis and toxicity in normal CXCR4^+^ tissues [236].

In a different approach, anti-CXCR4 antibodies were conjugated to gold nanoparticles aiming to enable tumor-selective radiotherapy in CXCR4^+^ breast cancer cells. Generated antibody-gold nanoconjugates selectively internalized into tumor cells, increasing radiosensitization via oxidative stress and DNA damage upon ionizing radiation, leading to significant tumor growth inhibition in vivo [237]. Similarly, superparamagnetic iron nanoparticles decorated with anti-CXCR4 antibodies were used for targeted magnetic hyperthermia treatment, achieving a complete loss of cell viability in vitro, especially when combined with additional non-targeted magnetic particles to enhance total iron loading [238]. Another innovative approach involved the use of anti-CXCR4 antibodies to decorate doxorubicin-loaded quantum dot nanoparticles. This strategy allowed the targeted delivery and pH-controlled drug release into CXCR4^+^ myeloma cells in vitro providing a precision drug delivery mechanism [239].

Finally, a novel strategy fused the single-chain variable fragment (ScFv) of an anti-CXCR4 antibody to an RNA-binding protamine peptide to electrostatically couple macrophages polarizing miRNAs. These nanoplexes, targeted both tumor cells (through CXCR4 antagonism) and macrophages (via miR-127 delivery) to modulate the tumor microenvironment. This strategy successfully induced CXCR4^+^ macrophages polarization into M1 tumor-suppressive phenotype in a mouse model of triple-negative breast cancer, offering a dual-targeted therapeutic effect [240].

#### CXCR4-targeted protein nanoparticles

While antibodies represent the most widely used protein carriers for receptors targeting, alternative protein-based nanovehicles have been also successfully developed in the last decades, offering significant advantages over traditional antibodies. One of the main strategies involves the use of modular proteins to achieve multiple functions, including targeting, within a single polypeptide. In this context, viral-mimetic nanoparticles that self-assemble through divalent cation coordination were developed enabling the multivalent presentation of the CXCR4-targeting peptide ligand T22, which enhances receptor selectivity and internalization capacity in target cells [54, 241, 242]. Then, in a similar approach to ADCs, this multivalent nanocarrier (T22-GFP-H6) and it humanized version (T22-HSNBT-H6) were chemically conjugated with different antitumoral drugs including oligo-floxouridine (5-FdU) [111, 243], oligo-cytarabine (5-AraC) [244] or Monomethyl Auristatin E (MMAE) [245–248]. These nanoconjugates, when administered intravenously, selectively targeted CXCR4^+^ cancer cells, inducing their selective depletion and regression of stablished metastases in colorectal cancer models [111] and a potent blockade of tumor dissemination in mice models of AML [244, 245] and diffuse large B-cell lymphoma (DLBCL) [247].

Building on this technology, the biologically neutral GFP or HSNBT scaffolds were replaced with biologically active domains to create intrinsically therapeutic nanocarriers that directly exert their biological action upon receptor-mediated internalization. In this sense, the active proteins incorporated in this system included pro-apoptotic domains [249], insect venoms [250] and bacterial [251, 252] or plant toxins [253]. Among them, nanoparticles carrying the catalytic domain of *Pseudomonas aeruginosa* exotoxin (T22-PE24-H6) or *Corynebacterium diphtheriae* exotoxin (T22-DITOX-H6) showed the most potent and selective destruction of CXCR4^+^ cells via gasdermin-mediated pyroptosis [251, 252, 254–262].

Based on this literature, another group explored the use of the T22-PE24-H6 nanotoxin to induce tumor-selective and gasdermin-mediated immunogenic cell death in CXCR4^+^ melanoma mouse models, achieving strong synergy when combined with an anti-PD-1 immune checkpoint inhibitor therapy [263]. Then, expanding on the immunotherapy strategy, CXCR4-targeted immunostimulatory nanoparticles incorporating inflammatory proteins such as Gasdermin D (GSDMD) or Mixed Lineage Kinase Domain-Like protein (MLKL) were developed to induce tumor-selective pyroptotic cell death and subsequent activation of the immune system. Thus, in vivo administration of T22-GSDMD-H6 or T22-MLKL-H6 nanoparticles induced significant lymphocyte infiltration and tumor size reduction without associated toxicity [264].

Modular peptide nanocarriers have been also engineered to incorporate an SDF-1 derived peptide for CXCR4-targeting and a poly-arginine domain for the electrostatic condensation of RNAs, looking for targeted delivery of iRNAs into CXCR4^+^ cells. This system successfully delivered an anti-VEGFA siRNA into CXCR4^+^ endothelial and glioblastoma cells, reducing VEGF secretion and inhibiting endothelial cells migration, validating this strategy as a promising anti-angiogenic therapy [265].

Finally, Bovine Serum Albumin (BSA) has been also adapted as protein carrier for targeted drug delivery to CXCR4^+^ cells. In this case, the CXCR4 antagonist AMD3100 was chemically conjugated to BSA first, using a bifunctional linker, and paclitaxel-loaded nanoparticles were generated then via biomineralization process. The resulting BSA-PTX nanoparticles selectively internalized into CXCR4^+^ ovarian cancer cells, showing excellent biodistribution and significant inhibition of tumour growth and metastasis in vivo by a dual drug delivery and CXCR4-blocking mechanism [266].

## Conclusions and future perspectives

CXCR4 has emerged as a remarkable molecular target in cancer therapy, due to its overexpression in many types of tumors and its strong association with poor prognosis and cancer stem cell phenotype. Numerous tools have been developed to block and target this receptor for therapeutic use (Table 1), being most of them initially identified for its role in HIV infection. Over the last decade, many of these targeting strategies have been repurposed for cancer therapy, unlocking new possibilities for clinical interventions.

Given that CXCR4 is overexpressed in more than 20 different types of tumors, many of the molecules previously identified have now been applied to both cancer diagnosis and treatment through CXCR4 inhibition. These therapies have shown highly promising results, leading to numerous clinical trials (Table 2), particularly in combination with standard chemotherapies. However, while CXCR4-targeted treatments have improved the outcomes for CXCR4^+^ cancers, small molecule inhibitors and antibodies targeting CXCR4 signalling have yet to reach the market for cancer therapy. In this sense, the limited activity and the high toxicity observed in clinical trials, have in general hindered their clinical translation.

In this context, identifying effective molecular markers to distinguish between tumour and healthy cells is crucial to develop precision cancer therapies. Thus, targeted drug delivery to cancer cells has the potential to revolutionize the future of cancer medicine by improving the selective uptake of drugs in cancer tissues, thereby increasing therapeutic efficacy while minimizing systemic side effects. In this context, over the last decade numerous biotechnological approaches have emerged, designed to selectively deliver innovative therapies to CXCR4^+^ cancer cells. Their payloads include cytotoxic, anti-mitotic or anti-angiogenic agents, small interfering nucleic acids, photothermal molecules, or even radioactive isotopes for radiotherapy (Table 3). Thus, by concentrating these powerful agents in CXCR4 overexpressing tumour cells, researchers aim to maximize therapeutic outcomes while reducing collateral damage to healthy tissues. Another promising strategy involves the development of bispecific or multispecific drugs, such as antibodies or nanoparticles, which can simultaneously target multiple markers, including CXCR4 and other cancer stem cell markers. This strategy could offer a more comprehensive approach for addressing the plasticity of CSCs, which may evade treatment if only one marker is targeted. For instance, bispecific antibodies, like those targeting LGR5 and EGFR for colorectal cancer, could potentially improve outcomes by better controlling CSC populations and preventing neoplastic progression.

All in all, although many of these targeted delivery strategies are still in the preclinical phase, their promising results suggest that some of these biotechnological innovations may soon enter clinical trials. In this sense, the development of basket clinical trials focusing on patients with high CXCR4 expression, independent of tumor type, could further accelerate the adoption of these therapies. Such trials would selectively recruit patients with CXCR4^+^ cancers, thereby increasing the likelihood of positive responses while minimizing the risk of systemic toxicity. In this scenario, precision medicine will play a pivotal role in advancing CXCR4-targeted therapies. For that, imaging agents, such as radiotracers like Pentixafor, which can detect CXCR4 expression in tumors and metastases, will be invaluable in identifying patients most likely to benefit from these therapies, while monitoring their response to therapy. Thus, by combining these diagnostic tools with targeted drug delivery systems, it is expected to enhance both the efficacy and safety of CXCR4-targeted treatments, ultimately improving the therapeutic outcomes.

## Data Availability

No datasets were generated or analysed during the current study.
